# Diabetic Microvascular Disease and Pulmonary Fibrosis: The Contribution of Platelets and Systemic Inflammation

**DOI:** 10.3390/ijms17111853

**Published:** 2016-11-08

**Authors:** Rekha Jagadapillai, Madhavi J. Rane, Xingyu Lin, Andrew M. Roberts, Gary W. Hoyle, Lu Cai, Evelyne Gozal

**Affiliations:** 1 Department of Pediatrics, School of Medicine, University of Louisville, Louisville, KY 40292, USA; rekha.jagadapillai@louisville.edu (R.J.); 15943075920@163.com (X.L.); andrew.roberts@louisville.edu (A.M.R.); lu.cai@louisville.edu (L.C.); 2 Medicine/Nephrology, School of Medicine, University of Louisville, Louisville, KY 40292, USA; madhavi.rane@louisville.edu; 3 Biochemistry and Molecular Biology, School of Medicine, University of Louisville, Louisville, KY 40292, USA; 4 Department of Thoracic Surgery, the First Hospital of Jilin University, Changchun 130021, China; 5 Physiology, School of Medicine, University of Louisville, Louisville, KY 40292, USA; 6 Department of Environmental and Occupational Health Sciences, School of Public Health and Information Sciences, University of Louisville, Louisville, KY 40292, USA; gary.hoyle@louisville.edu; 7 Pharmacology & Toxicology, School of Medicine, University of Louisville, Louisville, KY 40292, USA; 8 Radiation Oncology, School of Medicine, University of Louisville, Louisville, KY 40292, USA

**Keywords:** lung fibrosis, endothelial injury, platelet, nitric oxide, JAK/STAT

## Abstract

Diabetes is strongly associated with systemic inflammation and oxidative stress, but its effect on pulmonary vascular disease and lung function has often been disregarded. Several studies identified restrictive lung disease and fibrotic changes in diabetic patients and in animal models of diabetes. While microvascular dysfunction is a well-known complication of diabetes, the mechanisms leading to diabetes-induced lung injury have largely been disregarded. We described the potential involvement of diabetes-induced platelet-endothelial interactions in perpetuating vascular inflammation and oxidative injury leading to fibrotic changes in the lung. Changes in nitric oxide synthase (NOS) activation and decreased NO bioavailability in the diabetic lung increase platelet activation and vascular injury and may account for platelet hyperreactivity reported in diabetic patients. Additionally, the Janus kinase/signal transducer and activator of transcription (JAK/STAT) pathway has been reported to mediate pancreatic islet damage, and is implicated in the onset of diabetes, inflammation and vascular injury. Many growth factors and diabetes-induced agonists act via the JAK/STAT pathway. Other studies reported the contribution of the JAK/STAT pathway to the regulation of the pulmonary fibrotic process but the role of this pathway in the development of diabetic lung fibrosis has not been considered. These observations may open new therapeutic perspectives for modulating multiple pathways to mitigate diabetes onset or its pulmonary consequences.

## 1. Diabetic Lung Fibrosis

Diabetes mellitus (DM) is a common chronic disease that exacts a huge toll on the health of the U.S. populace. As estimated by the Center for Disease Control in 2014, 29.1 million people, or 9.3% of the total U.S. population, and 12.3% of people who are 20 years or older, have diabetes [1]. Type 1 DM (T1DM) is usually diagnosed in children and young adults and comprises 5%–10% of the number of people with diabetes. The risk of long-term diabetic complications in T1DM is high because of the potential for hyperglycemia throughout the lifetime of affected individuals. Type 2 DM (T2DM) that comprises the remainder, typically develops later in life, is associated with obesity, and is expected to increase in prevalence in the coming years. Chronic metabolic abnormalities due to insulin deficiency or resistance, along with hyperglycemia, cause systemic oxidative stress and inflammation and impair antioxidant defense, leading to cardiovascular dysfunction [2,3,4,5]. These processes may underlie the development of fibrosis, reported in several clinical studies, that can occur in multiple organs, including the heart, kidneys, and lungs [2,5,6,7,8,9,10]. In addition to glycation of hemoglobin affecting oxygenation, chronic hyperglycemia may cause non-enzymatic glycation of the chest wall and bronchial collagen protein, leading to fibrotic processes and reduced ventilatory function [11]. However, detailed mechanisms of these fibrotic processes have not been elucidated, and treatments which can prevent or reverse organ dysfunction in diabetes are lacking. We and others previously reported that pulmonary functional capabilities are altered in type 1 and type 2 diabetes patients and that restrictive pulmonary dysfunction and fibrosis may be associated with diabetic complications [4,7,10].

Diabetes is strongly associated with systemic cardiovascular disease [2,3,12,13,14,15] but the relationship with pulmonary vascular disease has often been disregarded [16]. Pulmonary complications, while often subclinical in diabetic patients because of the lungs’ functional reserve, may become clinically important as patients subsequently develop chronic restrictive and obstructive pulmonary functional changes. Such changes would compromise ventilation and gas exchange, which together with endothelial dysfunction and excessive platelet activation may promote the induction of the fibrotic process [17,18,19]. Several studies identified a restrictive pattern in the lungs of diabetic patients that differed from smoking-induced lung pathology and was not correlated with confounding factors such as Body Mass Index (BMI), smoking, duration of the disease or HbA1c levels [10,20]. A comprehensive study, examining a full patient cohort of a large health care company, including T1DM and T2DM patients, identified an increased risk of lung disease including asthma, chronic obstructive pulmonary disease (COPD), fibrosis and pneumonia in DM patients [4]. This study reported that patients with a diagnosis of diabetes had a significantly higher risk of pulmonary fibrosis (heart rate (HR) and 95% CI (confidence interval), DM 1.54 (1.31–1.81)), compared to non-diabetic patients, and adjusted for age, sex, race, smoking, BMI, education, alcohol consumption, and number of outpatient visits [4]. Transbronchial biopsies performed on 12 DM patients and compared to 12 age and gender matched controls showed narrowed alveolar spaces with hyperplastic interstitium, collagen accumulation and fibrotic changes with inflammatory cells infiltration in the diabetic patients [10]. Other studies showed reduced lung diffusing capacities, also affecting peripheral circulation and vasoreactivity, in aerobically fit athletes with T1DM that could be remediated by careful glycemic control [21,22]. Furthermore, long-standing T1DM was associated with reduced lung volume and decreased alveolar capillary gas exchange [23]. Decreased pulmonary capillary blood flow was associated with the duration of diabetes in T1DM patients [24]. Multiple clinical studies established an association between T1DM and T2DM and lower values of the forced expiratory volume in 1 s (FEV1), vital capacity (VC) and pulmonary diffusion capacity to carbon monoxide (DLCO) [18]. A postmortem study comparing histological findings throughout the lung parenchyma of 6 controls and 6 diabetic patients (3 T1DM and 3 T2DM) reported the presence of macro- or microangiopathy and thickening of the lung basal lamina in the diabetic patients [5]. This occurred with homogenously and significantly increased alveolar epithelial basal lamina thickness in DM patients vs. controls (DM: 176 ± 27 nm vs. C: 121 ± 11 nm) and increased endothelial capillary basal lamina thickness (DM: 223 ± 27 nm vs. C: 164 ± 14 nm) [5]. Overall, these clinical findings suggest that increased attention should be granted to pulmonary dysfunction and fibrosis in diabetic patients that may require preventive and therapeutic interventions. These clinical findings were supported by several reports of pulmonary changes in diabetic animal models [7,8,10]. We showed significant fibrotic changes in the lungs of streptozotocin-injected FVB mice, 3 months after the onset of diabetes. These findings were confirmed 3 and a half months after the onset of the disease in the lungs of a different mouse diabetes model, OVE26 mice, that spontaneously develop T1DM two weeks after birth [7,10].

Impaired lung function is characterized by abnormal pulmonary vascular responsiveness, microvascular dysfunction, platelet activation, and lung fibrosis [8,25,26,27]. Microvascular injury is a well-known complication of diabetes that is likely to target multiple organs and contributes to the development of cardiac and pulmonary fibrosis. Pulmonary and cardiac blood flows are regulated by the endothelial cell layer, which forms a dynamic barrier between the underlying smooth muscle cells and circulating blood [28,29,30,31,32]. Diabetes-induced cardiac fibrosis has been extensively studied in both human and animal models [33,34,35,36]. In contrast, despite emerging clinical reports of diabetes-induced pulmonary fibrosis and microvascular damage in T1DM and T2DM patients, diabetes-induced pulmonary fibrosis has been poorly studied, and few studies have considered it independently from heart failure [16,20]. Susceptibility of diabetic individuals to fibrosis in multiple organs suggests the presence of widespread chronic injury underlying these complications. Whether this injury, or the response to injury leading to fibrosis, is similar in different organs is not known. Therefore, mechanisms leading to diabetes-induced lung injury and pulmonary microangiopathy, which compromise gas exchange and maintenance of a low pulmonary vascular resistance, need to be elucidated to improve our understanding and develop new treatment modalities. We reviewed the available data to provide an integrative vision of the combinatorial effect of diabetes-induced lung microvascular disease, platelet hyperreactivity and inflammation that are all known to contribute to the development of lung fibrosis. In addition, we attempted to identify signaling mechanisms shared by these factors that could contribute to the development of diabetes-induced lung fibrosis and may help to identify novel therapeutic options.

## 2. A Role for Platelets in the Pathology of Diabetes-Induced Microvascular and Lung Disease

**Figure 1:**
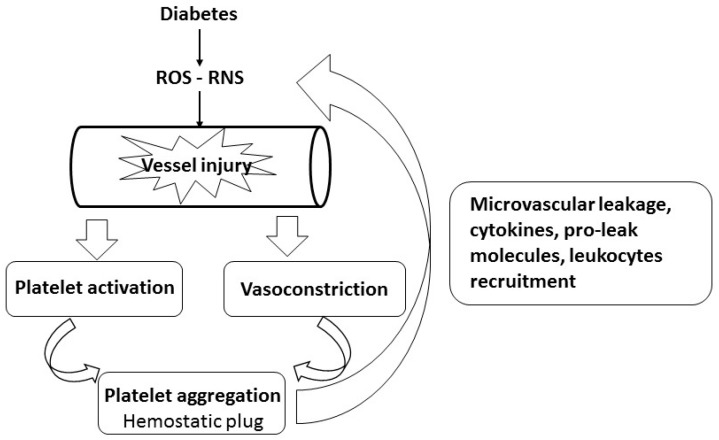
Schematic representation of diabetes-induced platelet involvement in vascular injury. Production of reactive oxygen and nitrogen species (ROS and RNS) in diabetic subjects causes vascular injury, which in turn induces vasoconstriction, platelet activation and aggregation and release of platelet granules content. These events enhance vascular oxidative injury, inflammation and microvascular leakage.

The concept of diabetes-induced pulmonary microvascular dysfunction is well recognized [17,27], but changes in the pulmonary microenvironment likely to cause pro-thrombotic responses via interactions between platelets, blood flow and the vascular endothelium have not been well characterized [37]. Platelets play a significant role in acute lung injury in initiating inflammation, increasing microvascular leakage, and promoting ventilation/perfusion mismatch [38,39,40]. They contribute to redox imbalance through production of reactive oxygen species (ROS) and reactive nitrogen species (RNS), pro-leak molecules, and recruitment of inflammatory cytokines and leukocytes to the damaged endothelium (Figure 1) [28,39,40,41,42,43]. The lungs are a significant source of megakaryocytes, generating platelets that contribute to basal barrier integrity of the alveolar capillaries and to pulmonary vascular repair [19,39].

**Figure 2:**
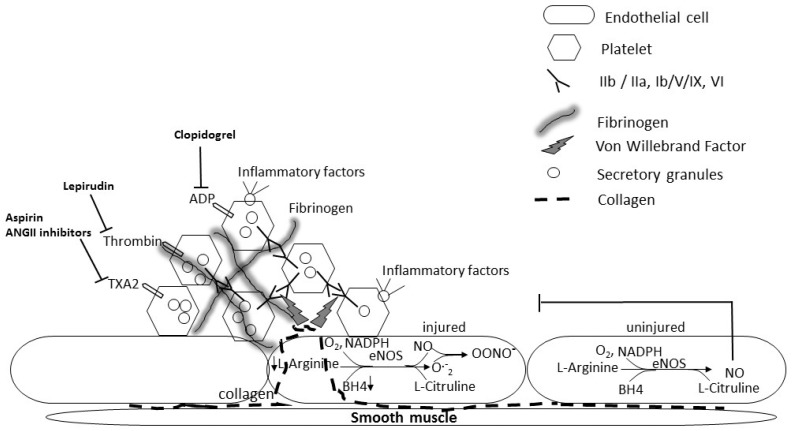
Platelet aggregation at the site of vascular injury. Sub-endothelial collagen exposed by injury binds to von Willebrand factor (vWF), which in turns binds to platelets via GPIb-IX-V platelet complex, increasing GPVI and α2β1integrin binding to collagen. Bound platelets become activated, and bind tightly to adhesion proteins including vWF, fibrinogen and fibronectin. Activated platelets then release preformed granule contents with vasoconstrictors and inflammatory mediators as well as ADP, thromboxane (TXA2) and thrombin, inducing autocrine paracrine platelet activation. The interactions of these factors with platelets can be inhibited by Clopidogrel, Lepirudin, aspirin and other inhibitory compounds. Additionally, NO produced in intact endothelium by endothelial NOS (eNOS) inhibits platelet activation. However eNOS becomes uncoupled in injured endothelium and yields superoxide (O_2_^•−^) instead of NO. O_2_^•−^ reacting with iNOS-produced NO forms peroxynitrite (OONO^−^), enhancing endothelial injury and platelet involvement.

Platelets play a role in supporting the function and integrity of the resting vascular endothelium, promoting endothelial cell growth, blocking gaps in the endothelial layer and releasing factors enhancing the endothelial barrier function (reviewed in [40]). However, platelets also contribute to injury and to leukocyte recruitment and infiltration through their interaction with activated endothelial cells in a variety of pulmonary disorders [19,39,43,44,45]. Platelets store and secrete various cytokines, such as transforming growth factor beta (TGF-β) or platelet-derived growth factor (PDGF), and are implicated in the development of fibrosis and in various forms of idiopathic pulmonary fibrosis with platelet dysfunction or platelet granules abnormalities [19]. Platelet hyperreactivity in diabetic patients has been reported as early as 1965 and is associated with hyperglycemia, hyperlipidemia, inflammatory and oxidant states and increased glycation of surface membrane proteins, as well as higher calcium and lower cAMP levels that increase platelet sensitivity to agonists [37,46,47,48,49,50,51,52]. The osmotic effect of glucose may also increase platelet reactivity [37,50]. Both insulin deficiency as in T1DM and insulin resistance as in T2DM, increase platelet reactivity [37,51,52,53]. Newly diagnosed insulin-dependent patients showed increased levels of large circulating activated platelets [53]. Platelet activation evidenced by increased P-selectin and other soluble markers of platelet activation, and characterized by impaired calcium homeostasis, oxidative stress and upregulation of P2Y_12_ signaling, could be mitigated by improved glycemic control [52]. Dyslipidemia, obesity and systemic inflammation associated with T2DM may also contribute to platelet hyperreactivity. A clinical study of platelet inhibition in 157 cardiovascular disease patients treated with the platelet inhibitor clopidogrel, revealed a lower response to the drug in DM patients and in patients with high BMI (≥25 kg/m^2^) and a significant interaction between DM and elevated plasma fibrinogen [53,54,55]. Fibrinogen mediates platelet plug formation and stabilizes clots through polymerization of fibrin produced by thrombin cleavage of fibrinogen that are elevated in DM patients [37,52]. Upon inflammatory conditions, upregulation of fibrinogen recruits inflammatory cells and platelets, activating endothelial cells and contributing to persistent vascular inflammation [19,39,40,56]. Endothelial cells are attached to the subendothelial collagen by von Willebrand factor (VWF), which they produce. When the endothelial layer is disrupted, collagen is exposed and VWF binds to collagen and anchors platelets to the subendothelium by binding platelet GP1b-IX-V receptor, causing platelet aggregation and formation of a platelet plug (Figure 2) [19]. In addition to fibrinogen’s role in the clotting cascade, it is involved in normal and abnormal clotting and inflammatory responses and may induce platelet thrombogenesis and alterations in vascular reactivity (Figure 2) [39,57,58]. Studies using other platelet inhibitors such as aspirin regimens, showed similar suboptimal platelet inhibition in DM patients [47]. Recent studies in mice and other animal models of diabetes showed that platelets increase alveolar capillary permeability and identified serotonin from platelets as a mediator that links vascular damage to extravascular tissue fibrosis [59]. Therefore, platelets play a role as effector cells in pulmonary vascular diseases and in chronic pulmonary vascular syndromes [39,56] and may contribute to DM-induced lung fibrosis. However, these pulmonary effects of fibrinogen and the mechanism of platelet-mediated microvascular dysfunction in diabetic lung have largely been disregarded. Platelet aggregation, activating coagulation signaling cascades, influences key inflammatory and fibroproliferative responses resulting in the development of fibrosis [56]. Although glycemic control has been shown to decrease platelet hyperreactivity [37,52], no study examined the effect of platelet inhibition on DM-induced pulmonary fibrosis. The effect of inhibiting platelet activation with such inhibitors as clopidogrel (ADP receptor P2Y12 inhibitor) or eptifibatide (that prevents αIIbβ3-mediated platelet aggregation) on the development of DM-induced lung fibrosis, when administered to DM patients or DM animal models at their increased effective dosage, should be examined. Additionally, despite limitations mainly due to species differences, new animal models for in vivo generation of genetically modified platelets are being developed that may provide new tools to manipulate platelet signaling [60]. Platelet-specific knock out (KO) or knock-in animals may allow us to modulate platelet function and determine the effect of platelet-induced cascades on diabetes and diabetes-induced lung fibrosis.

## 3. Putative Signaling Mediating Diabetes-Induced Microvascular Disease and Lung Fibrosis

**Figure 3:**
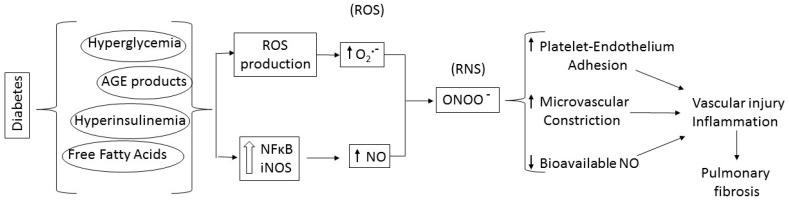
Schematic representation of diabetes-induced pathways to pulmonary fibrosis. Diabetes-induced pathological changes generate reactive oxygen species (ROS) such as superoxide (O_2_^•−^) and activate inflammatory pathways such as NF-κB-dependent genes and increased iNOS production, releasing NO, which reacts with excessive O_2_^•−^ to form reactive nitrogen species (RNS) such as peroxynitrite (OONO^−^). This chain reaction contributes to increased vasoconstriction, platelet-endothelium adhesion, and decreased bioavailable NO, thereby propagating vascular inflammation. Inflammation combined with the oxidative environment causes fibrosis.

The involvement of reactive oxygen and nitrogen species (ROS and RNS) in diabetic pulmonary fibrosis has been reported in mice [7,29,61]. Diabetes detrimental systemic effect, with excessive NO combined with ROS, may directly contribute to platelet activation and cause chronic inflammation associated with damaged lung capillary endothelium and microangiopathy (Figure 3) [27,49,62,63]. Streptozotocin-induced diabetes caused pulmonary endothelial dysfunction in rats by enhancing NADPH oxidase-derived superoxide production, impairing dilation to acetylcholine and reducing vasoconstriction to inhibition of nitric oxide. Such physiological effects may explain a higher incidence of pulmonary arterial hypertension in diabetic patients [64].

We and others demonstrated the development of diabetic pulmonary fibrosis in mouse models of T1DM and showed that ROS, RNS and angiotensin II play roles in the development of these pathologies [7,64]. Additionally, activating antioxidant pathways by administration of zinc by gavage showed significant beneficial effects of preventing and reversing non-enzymatic glycation, oxidative injury and lung damage in streptozotocin-induced diabetic rats and in a T2DM mouse model [2,65]. Multiple clinical and animal studies reported renal and cardiac fibrosis associated with diabetes, which progressed to end-stage kidney disease or heart failure as major consequences of diabetes [3,6,13,66]. Nitrosative processes are implicated in these pathologies, as well as in diabetes-induced endothelial dysfunction that may underlie DM-induced pulmonary fibrosis [13,29,66,67,68].

Recent studies reported reduced basal NO release and significantly increased superoxide levels in mesenteric and tail arteries of streptozotocin-induced diabetic rats that impaired NO-dependent and EDHF-dependent relaxation [69,70]. Similar decreases in NO bioavailability resulting in impaired endothelial function were also shown in T2DM patients that could be improved by stimulating autophagy with compounds such as spermidine [71]. These authors reported that inhibiting autophagy in endothelial cells from healthy patients resulted in a similar decrease in bioavailable NO, as seen in diabetic patients’ cells, suggesting that inadequate autophagy may contribute to diabetic endothelial dysfunction.

NO is enzymatically synthetized from l-arginine and molecular oxygen by NO synthase (NOS) and plays a critical role in the regulation of pulmonary circulation during physiological as well as pathophysiological circumstances [29,30,32]. Three known NOS isoforms have been identified in the lung that can play a role in the pulmonary circulation: the endothelial (eNOS) and the neuronal (nNOS) isoforms, regulated by calcium and calmodulin, and the inducible isoform (iNOS) that can be induced by inflammatory stimuli (reviewed in [72,73]). The constitutive eNOS, expressed by vascular endothelial cells, has a central role in modulating vascular tone, inhibits platelet aggregation, and can be altered by various forms of endothelial cell injury [29,30,32,72]. eNOS-derived NO, in response to chemical release (bradykinin, acetyl choline or calcium ionophore) or due to physical stress, induces vasodilation, reducing vascular resistance and blood pressure, and inhibits platelet and leukocyte adhesion and aggregation [29].

**Figure 4:**
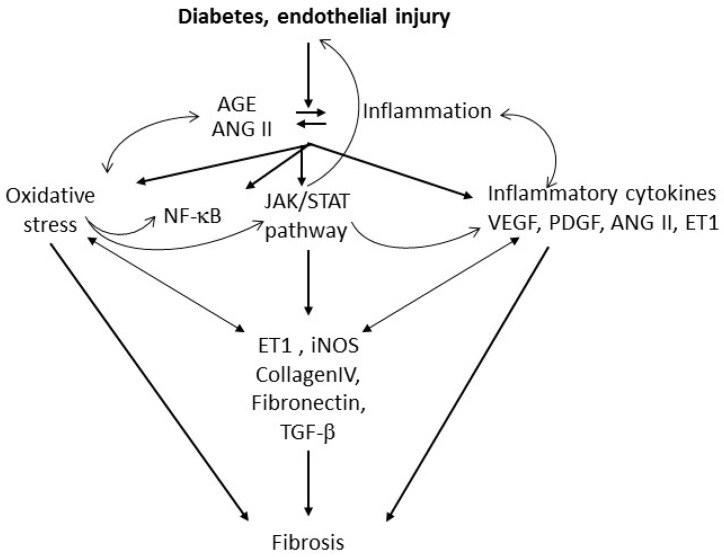
Overview of signaling pathways contributing to diabetes-induced pulmonary fibrosis. Interdependent and bilateral interactions between multiple pathways contribute to enhance injury and stimulate the fibrotic response. VEGF: vascular endothelial growth factor; PDGF: platelet-derived growth factor; ANG II: angiotensin II; ET1: endothelin-1.

Excessive NO may out-compete antioxidants for ROS scavenging, forming secondary RNS products, such as nitrosonium cation (NO^+^), nitroxyl anion (NO^−^), and peroxynitrite (ONOO^−^). These RNS products may also play a role in the development of fibrosis and modulate platelet function causing additional oxidative stress, microvascular dysfunction and injury [31,32,64,72,74]. However, NO reactivity with ROS may result in decreased NO bioavailablity, and vasoconstriction adding to the endothelial injury caused by toxic NO derivatives. Therefore in the presence of excess superoxide in the diabetic lung, bioavailable NO may be depleted, affecting vascular tone and endothelial function, while production of toxic reactive nitrogen species causes vasoconstriction and enhanced endothelial injury (Figure 3) [28,29,30]. Lack of endogenous NO may also contribute to platelet and leukocyte-induced injury, since NO has anti-inflammatory effects. NO at physiological concentrations has a protective role, inhibiting platelet aggregation, integrin-mediated adhesion and induction of pro-inflammatory genes. In contrast, at high concentration and in the presence of oxidative species, NO can form RNS and become deleterious [74]. NO influences major aspects of platelet functions including calcium mobilization, shape change, secretion and integrin activation and decreased ADP availability [31]. Disruption of eNOS or nNOS genes or nonselective NOS inhibition may cause insulin resistance in normal rodents [75]. iNOS disruption reverses high fat stimulated insulin resistance in mice or in *ob*/*ob* mice [76,77]. Therefore, although all NOS isoforms generate NO, the effects of inducible and constitutive NOS on insulin resistance appear to diverge, as described in diseases such as heart failure, hypertension and stroke [67,68]. Of note, pulmonary iNOS expression was increased in eNOS knockout mice, suggesting a cross-regulation between the two isoforms [78]. Indeed, overexpression of iNOS, and formation of peroxynitrite influences the regulation of the NOS isoforms eNOS and iNOS and the ability of NO to regulate its own production [31]. Endothelial disruption with leukocyte and platelet adhesion is expected to result in loss of endothelium-derived NO, damage to the pulmonary endothelium and impaired endothelium-dependent vasodilation, favoring platelet-induced vasoconstriction [79]. Studies showed increased eNOS monomerization, altering enzymatic function (eNOS uncoupling) and resulting in increased production of superoxide in diseases such as diabetes and atherosclerosis (Figure 2) [80]. iNOS, originally identified in platelets and inflammatory cells such as neutrophils and macrophages, is widely distributed in the endothelium and vascular smooth muscle [72,74,81,82]. iNOS expression is upregulated by many inducers of insulin resistance such as obesity, free fatty acids, hyperglycemia and oxidative stress (reviewed in [68]). Platelets of T2DM patients showed elevated levels of iNOS and increased production of peroxynitrite [83]. iNOS-derived NO modulates inflammation and regulates immune responses through various pathways. Whether induction of iNOS is beneficial or detrimental depends on the type of insult, the level and duration of expression, and the redox status of the tissue. Overall, decreased NO bioavailability with increased production of reactive nitrogen species as reported in animal and human studies of diabetes, supports a role for platelets in diabetes-induced endothelial and pulmonary injury (Figure 3). Studies in our laboratory have previously shown that genetically-induced antioxidant pathways, such as metallothionein (MT) or Nrf 2 (nuclear factor erythroid-related factor 2) overexpression, or pharmacological induction of these pathways by zinc administration (to induce MT) or sulforaphane (to induce Nrf2) alleviate DM-induced cardiac inflammation oxidative stress and fibrosis [2,84,85,86,87,88,89]. Ongoing similar studies are in progress in our laboratory to determine the role of this pathway in inducing platelet hyperreactivity and DM-induced lung fibrosis. The use of cardiomyocyte-specific MT and Nrf2 transgenics that were shown to alleviate DM-induced cardiac fibrosis will allow us to determine whether lung fibrosis occurs independently from cardiac fibrosis, should these mice still develop lung fibrosis. Furthermore, we will examine whether inhibiting platelet activation in diabetic mice with clopidogrel or eptifibatide will prevent the decrease in NO bioavailability in the diabetic lung and significantly attenuate lung inflammation and oxidative markers. In addition, iNOS and eNOS-deficient mice are commercially available and could be used to confirm the effect of modulating the various NOS isoforms signaling on DM-induced platelet hyerreactivity and pulmonary fibrosis. Experiments should be conducted to determine whether eNOS overexpression could prevent DM-induced decrease in NO bioavailability and modulate DM-induced lung fibrosis. Cardiac consequences of DM have been shown to be alleviated by genetic modulation of iNOS and eNOS. Therefore, using cardiomyocyte-specific genetically modified mice could shed some light on the contribution of DM-induced cardiomyopathy to platelet hyperactivity and lung fibrogenesis. STAT3 is a latent cytoplasmic transcription factor that can couple with multiple cytokines and growth factor receptors, such as gp130/IL6 receptor, VEGF (vascular endothelial growth factor), PDGF, as well as G-protein coupled receptor ligands such as angiotensin II and endothelin I (ET1). Together, this suggests that the JAK/STAT pathway plays an important role in inflammation and vascular injury (Figure 4). The human ET1 promoter contains a STAT1-gamma-activated sequence, and transient overexpression of STAT1, up-regulated ET1 promoter activity [90]. In contrast, JAK/STAT inhibitors significantly decreased high glucose-induced ET1 synthesis in a human endothelial cell line [90]. Moreover, JAK/STAT activation by ET1 was reported in endothelial cells and in vascular smooth muscle cells [91]. Therefore, the JAK/STAT signaling cascade may play a significant role in hyperglycemia-induced endothelial dysfunction in diabetes (Figure 4) [91,92].

There are 4 JAK (JAK 1–3 and Tyk2) tyrosine kinases and seven STAT proteins activated by specific cytokines. JAK 1/2 and Tyk2 are associated with the physiological response to gp130 signaling (reviewed in [93]). The system is inhibited by a number of proteins, notably suppressor of cytokine signaling (SOCS) proteins.

The JAK/STAT pathway contributes to β-cell dysfunction in both T1DM and T2DM [94]. It was recently reported that dysregulation of the JAK/STAT pathway contributes to the development of obesity and diabetes [94]. Targeted deletion of STAT3 in the brain in a rodent model leads to the development of obesity and diabetes mimicking the phenotype of *ob*/*ob* or *db*/*db* mice; however, these data have not yet been confirmed in human studies [95]. Interferon regulatory transcription factor-1, multiple chemokines, as well as iNOS are STAT1 target genes. Therefore, STAT1 could contribute to iNOS upregulation in the diabetic lung [96,97]. The pro-inflammatory role of the JAK/STAT pathway is well established in the macrophage [98,99,100]. Antioxidants such as resveratrol have been reported to inhibit inflammatory pathways in mouse macrophages by inhibiting JAK/STAT signaling pathways, suggesting that DM-induced oxidative stress in the lung, contributing to DM-induced lung inflammation, could be mediated via JAK/STAT activation [98]. A recent study reported that Sitagliptin, a dipeptidyl peptidase-4 inhibitor, improved diabetic cardiomyopathy and cardiac fibrosis induced by streptozotocin in rats through inhibition of JAK2 and STAT3 phosphorylation [101]. Studies in mouse models of bleomycin-induced lung fibrosis and CCl4-induced liver fibrosis suggested a protective role for STAT1 [102,103]. Another study in rats suggested that STAT1 inhibits bleomycin-induced lung fibrosis [104], while reduced SOCS1 expression has been linked to pulmonary fibrosis in patients and in animal models [105]. JANEX, a specific JAK3 inhibitor, prevented the development of autoimmune type 1 diabetes in NOD mice by modulating T cell cytokine profile [106]. Additionally, JANEX prevented cytokine-induced iNOS increase and NF-κB activation, restoring insulin secretion in cultured islets [107]. Therefore, JAK3 inhibition in the lung could similarly decrease the activation of the inflammatory cascade in the diabetic lung. AG490 and TG101348, two JAK2 specific inhibitors, have been shown to impair the JAK2/STAT3 signaling pathway in platelets, inhibiting platelet aggregation and activation in collagen-stimulated human platelets and platelet plug formation in a mouse model of irradiated mesenteric venules [108]. Furthermore, JAK3^−/−^ mice are protected from streptozotocin-induced diabetes and IL-1β and IFN-γ-induced islet damage [107]. These data suggest that STAT activation plays a significant role in pancreatic iNOS induction and the ensuing systemic inflammation in DM as well as in the fibrogenesis process [101,102,103,104,105]. Therefore, lung-targeted modulation of this pathway, could also prevent DM-induced pulmonary fibrosis by decreasing DM-induced platelet activation and lung inflammation. The contribution of this pathway to DM-induced platelet hyperactivation and endothelial injury is unknown and should be evaluated, using genetically altered mice or specific inhibitors in mouse models of diabetes. JAK/STAT inhibitors and mouse models with genetic deletion or overexpression of JAK/STAT pathway proteins have not been used to evaluate the therapeutic potential of modulating this pathway to alleviate DM-induced lung fibrosis. Thus, STAT1 and STAT3 appear to have divergent roles in the physiopathology of DM-induced lung fibrosis. While several JAK/STAT pathways inhibitors have been developed, the comprehensive effects of this pathway in multiple organs may limit the potential of this therapeutic approach and require the identification of more specific downstream targets.

## 4. Conclusions

Diabetes is a chronic disease associated with inflammation, oxidative stress, and endothelial injury and is implicated in significant cardiovascular and renal injury. Reports of pulmonary repercussions of diabetes have recently emerged; these are often characterized as subclinical, but may progress to significant restrictive and obstructive pulmonary pathologies with fibrotic changes in long-term DM patients. Improved therapeutic interventions in DM, which significantly prolong the lifespan of DM patients, may underlie the increase in reports of DM-induced lung injury. Platelet hyperreactivity in diabetic patients and disruption of NOS signaling combined with oxidative stress may mediate systemic endothelial injury, a hallmark of DM, and could represent major mediators in the development of diabetes-induced pulmonary fibrosis.

NOS activity is altered in DM and decreased NO bioavailability resulting from increased iNOS activity in an oxidative environment has been implicated in the development of renal and pulmonary fibrosis and could contribute to lung fibrosis (Figure 3). Furthermore, many growth factors and agonists involved in DM-induced lung fibrosis act via JAK/STAT activation that also regulates iNOS expression and platelet activation. This pathway appears to be important in the lung fibrotic response to diabetes (Figure 4). These pathways may interact in many ways and one pathway may be critically involved in one patient’s disease while the other more prominently acts on another patient. While multiple studies report successful therapeutic intervention with one or the other pathway, it is unlikely that modulation of a single pathway will lead to a comprehensive cure of DM or its consequences and a systemic approach may be required to achieve DM prevention and treatment.
